# Silent but Obstructive: A Left Atrial Myxoma Incidentally Identified During Cardiac Decompensation

**DOI:** 10.7759/cureus.87590

**Published:** 2025-07-09

**Authors:** Soufiane Touiti, Asmae Ouissaden, Tanaa El Ghali, Fatima Azzahra Benmessaoud, Nawal Doghmi, Cherti Mohamed

**Affiliations:** 1 Cardiology B Department, Mohammed V University of Rabat, Rabat, MAR

**Keywords:** cardiac mass tumor, heart failure, left atrial myxoma, mitral obstruction, pulmonary hypertension

## Abstract

Cardiac myxomas are rare benign tumors, most commonly located in the left atrium. Despite their benign histology, they may present with serious clinical consequences due to obstruction, embolism, or systemic symptoms. Their presentation can mimic valvular heart disease or heart failure, often delaying diagnosis. We report the case of a 72-year-old woman with a history of diabetes and hypertension who presented with progressive exertional dyspnea, New York Heart Association (NYHA) class III, evolving over two weeks. Clinical examination revealed signs of pulmonary congestion and pulmonary hypertension. Transthoracic echocardiography demonstrated a large, heterogeneous, mobile mass in the left atrium prolapsing into the mitral valve with a transmitral gradient of 14 mmHg and an estimated pulmonary artery pressure of 60 mmHg. Coronary angiography ruled out associated coronary artery disease. Surgical excision of the mass was performed successfully the following day. The postoperative course was uneventful, with resolution of symptoms. Histopathological examination confirmed the diagnosis of a left atrial myxoma with no features of malignancy.

This case highlights the obstructive presentation of a left atrial myxoma mimicking mitral stenosis, leading to elevated left atrial pressure and pulmonary hypertension. Echocardiography was essential for diagnosis and surgical planning, particularly given the risk of embolization and hemodynamic deterioration. Clinicians should maintain a high index of suspicion for structural cardiac tumors in elderly patients presenting with unexplained dyspnea and preserved ejection fraction. Early recognition and surgical management are essential to prevent serious complications and ensure favorable outcomes.

## Introduction

Cardiac myxomas are the most common primary tumors of the heart, accounting for approximately 50% of all benign cardiac neoplasms [1]. Despite their benign histological nature, they may have serious clinical consequences due to their strategic intracardiac location and potential for obstruction or embolization [2]. The reported incidence of primary cardiac tumors is rare, ranging from 0.0017% to 0.03% in autopsy series, with the majority arising in the left atrium [3]. These tumors may remain asymptomatic for long periods. Their clinical presentation depends largely on size, mobility, and location, and they can mimic various cardiac conditions, most notably mitral valve disease or heart failure [4]. Obstructive symptoms due to mitral inflow obstruction are one of the main mechanisms by which left atrial myxomas become clinically evident, especially when the tumor prolapses into the mitral valve during diastole. This can lead to elevated left atrial pressure, pulmonary hypertension, and progressive exertional dyspnea. Such presentations may easily be mistaken for more common causes of heart failure, delaying diagnosis [5]. We report a case of a left atrial myxoma revealed by acute pulmonary decompensation.

## Case presentation

**Figure 1:**
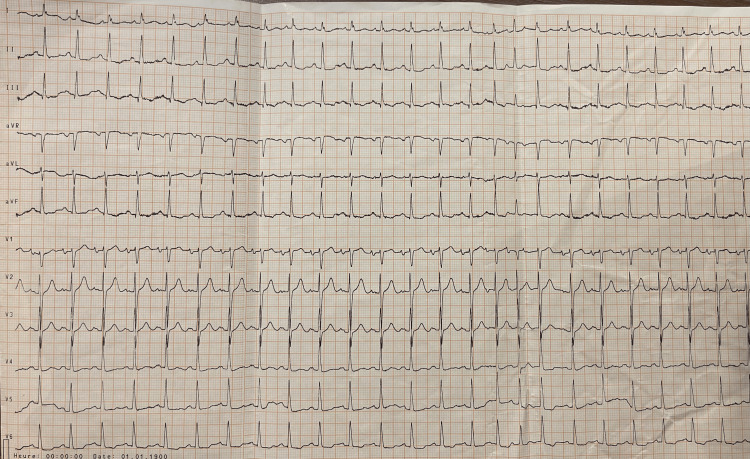
Sinus rhythm with a heart rate of approximately 90-100 bpm without repolarization abnormalities or conduction disturbances.

A 72-year-old woman with a medical history of type 2 diabetes mellitus and long-standing hypertension was admitted to our cardiology department for evaluation of worsening exertional dyspnea. The symptoms had progressed over two weeks from New York Heart Association (NYHA) functional class II to class III. She denied chest pain, palpitations, syncope, or fever. On physical examination, the patient was hemodynamically stable and eupneic at rest, with a heart rate of 106 beats per minute and normal blood pressure. Pulmonary auscultation revealed bibasilar crackles. Cardiac auscultation identified a loud second heart sound in the pulmonary area, suggestive of pulmonary hypertension. There were no signs of right-sided heart failure or peripheral congestion. A chest radiograph demonstrated an alveolo-interstitial pattern with hilar vascular congestion and peripheral redistribution, consistent with pulmonary venous hypertension. A 12-lead electrocardiogram revealed a normal sinus rhythm without repolarization abnormalities or conduction disturbances (Figure 1).

**Figure 2:**
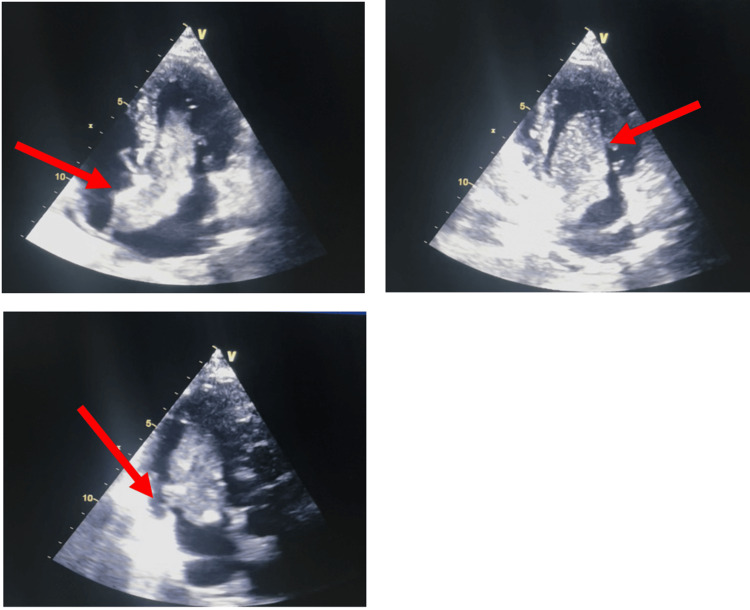
Transthoracic echocardiographic views (apical four-, two-, and three-chamber views showing a large, mobile heterogeneous mass in the left atrium, attached to the interatrial septum and prolapsing into the mitral valve during diastole. Top left (four-chamber view): A large, heterogeneous mass is seen arising from the interatrial septum, prolapsing into the left ventricle through the mitral valve during diastole. Top right (two-chamber view): The mass is clearly visualized within the left atrium, extending toward the mitral orifice, highlighting its mobility and risk of obstruction. Bottom (three-chamber view): The lesion appears heterogeneous and mobile, protruding into the left ventricular inflow tract, further supporting the diagnosis of a myxoma.

Transthoracic echocardiography showed a non-dilated, non-hypertrophied left ventricle with preserved global and segmental systolic function (left ventricular ejection fraction: 60%). A large, heterogeneous echogenic mass was identified in the left atrium, measuring approximately 70 × 30 mm, attached to the interatrial septum. The mass prolapsed through the mitral valve during diastole, causing a mean transmitral gradient of 14 mmHg. The estimated systolic pulmonary artery pressure was 60 mmHg, indicating significant pulmonary hypertension. These findings were consistent with an obstructive left atrial myxoma (Figure 2, Appendices), prompting surgical planning.

**Figure 3:**
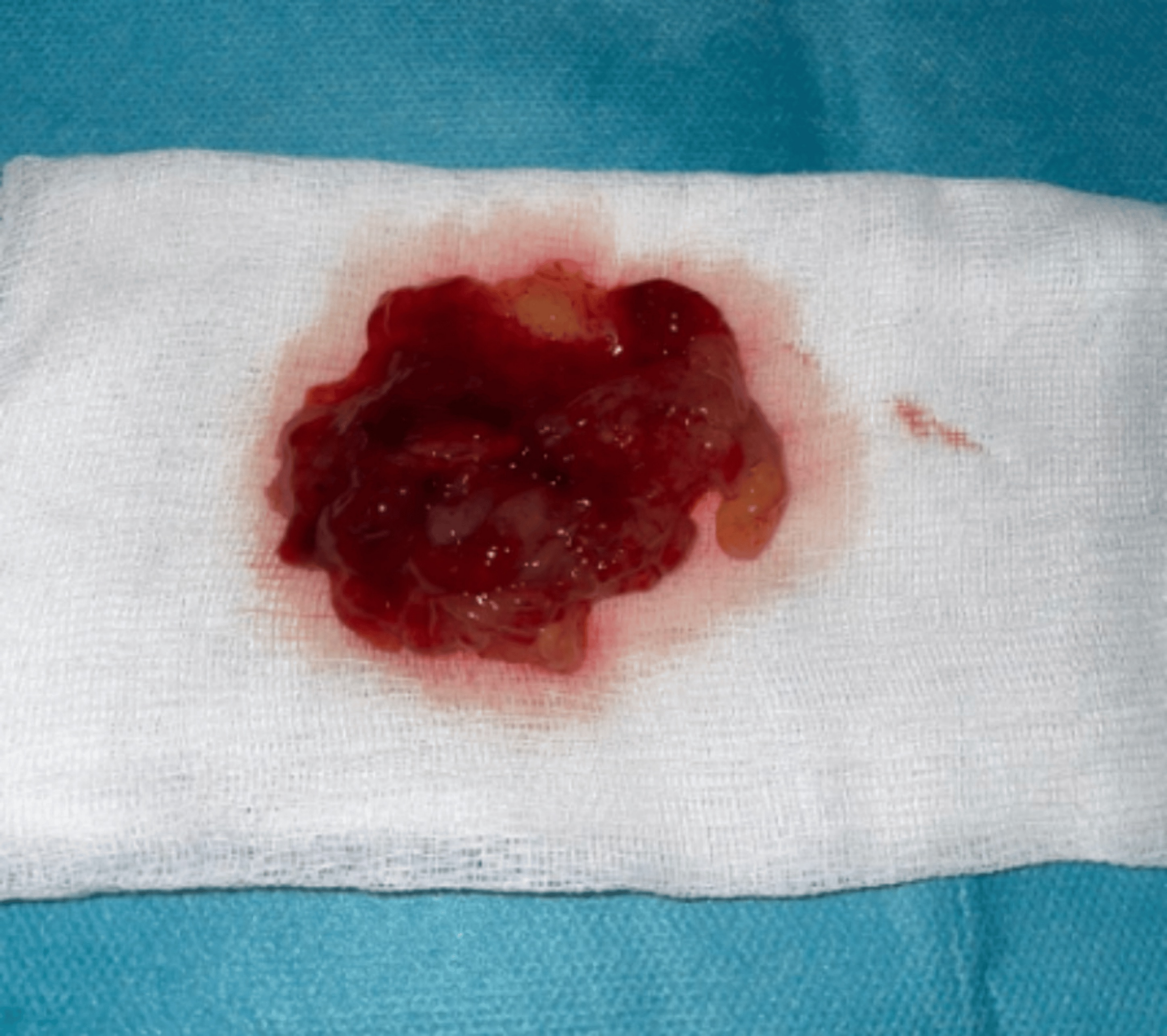
Macroscopic view of the surgical specimen showing a soft, lobulated, gelatinous mass with a white-grayish surface and hemorrhagic areas. These characteristics are consistent with a typical left atrial myxoma.

Initial management included intravenous diuretics to relieve pulmonary congestion and optimize the patient's volume status. Given the patient's age and cardiovascular risk profile, a preoperative coronary angiography was performed and demonstrated no significant coronary artery disease. Surgical excision of the intra-atrial mass was performed the following day via median sternotomy under cardiopulmonary bypass. The mass was successfully removed along with its septal attachment. The macroscopic examination of the surgical specimen revealed a soft, white-grayish mass measuring 6 cm in its largest dimension and weighing 53 grams (Figure 3).

The slight discrepancy between echocardiographic size and surgical measurement is likely due to tissue shrinkage after excision and fixation. An estimated systolic pulmonary artery pressure of 60 mmHg meets the echocardiographic definition of pulmonary hypertension (threshold ≥ 40 mmHg).

The postoperative course was uneventful. The patient was extubated within hours of surgery, remained hemodynamically stable, and was transferred out of the intensive care unit after 48 hours. Clinical improvement was marked, with complete resolution of dyspnea. Histopathological examination of the excised mass confirmed the diagnosis of left atrial myxoma, showing the typical features of a myxoid stroma with scattered stellate cells and spindle-shaped cells embedded in a mucopolysaccharide matrix and no evidence of malignancy. A transthoracic echocardiogram performed three days after surgery showed no residual mass or mitral inflow obstruction. The patient was discharged on day 5 and scheduled for annual echocardiographic follow-up to monitor for recurrence.

## Discussion

Cardiac myxomas are the most common primary cardiac tumors, accounting for nearly 50% of benign cardiac neoplasms [6]. Although histologically benign, their intracavitary location and potential for mechanical obstruction or embolization make them clinically significant. Most myxomas (approximately 75%) originate in the left atrium, often from the interatrial septum at the fossa ovalis [7]. Their presentation is highly variable and depends on the tumor’s size, location, and mobility. The classic clinical triad includes constitutional symptoms (fever, weight loss, fatigue), embolic events, and signs of intracardiac obstruction [8].

In our case, the patient presented with rapidly progressive dyspnea in the absence of chest pain, fever, or arrhythmia. The physical examination revealed signs consistent with pulmonary congestion and pulmonary hypertension, including bibasilar crackles and an accentuated second heart sound in the pulmonary area. These findings, coupled with a preserved left ventricular systolic function, prompted further evaluation to identify a mechanical cause of left-sided heart failure.

Transthoracic echocardiography identified a large left atrial mass causing dynamic mitral inflow obstruction and secondary pulmonary hypertension, clinically simulating mitral valve disease and illustrating a common source of diagnostic delay [9,10].

Echocardiography remains the cornerstone of diagnosis. Transthoracic echocardiogram (TTE) offers initial visualization, while transesophageal echocardiogram (TEE) provides superior resolution, especially when assessing the tumor's point of attachment and mobility. In this case, the echocardiographic features were strongly suggestive of a myxoma, and no differential diagnoses (such as thrombus or other masses) were retained [11].

Advances in multimodal imaging, including CT, MRI, and 3D reconstruction, have further refined pre-operative planning, especially for atypical or giant tumors [12,13]. However, in our cases, cardiac CT or MRI was not performed, as transthoracic echocardiography provided sufficient diagnostic clarity.

In elderly patients with cardiovascular risk factors, preoperative coronary angiography is recommended to evaluate for concomitant coronary artery disease, which could influence surgical planning. In our patient, the coronary angiogram was unremarkable, allowing isolated tumor excision.

Surgical resection is the definitive treatment and should not be delayed due to the risk of embolic events, sudden cardiac death, or worsening heart failure. Excision involves the removal of the mass along with its attachment base, often with patch repair of the septum. In our case, surgery was performed the day after diagnosis, with an uneventful postoperative course and rapid clinical improvement. No patch repair of the septum was needed during surgery, as no significant septal defect was observed. The histopathological analysis confirmed the diagnosis of a left atrial myxoma, showing typical myxoid stroma without features of malignancy.

Long-term prognosis following resection is excellent, with low recurrence rates (<3%) in sporadic cases [14]. However, recurrence is more frequent in familial forms or in the context of Carney complex, a rare syndrome associating myxomas, skin pigmentation, and endocrine tumors [15]. Evaluation for Carney complex was not deemed necessary given the patient's age, absence of pigmentation anomalies, endocrine abnormalities, or family history of cardiac tumors. As such, long-term follow-up with periodic echocardiography is advised to monitor for recurrence.

This case emphasizes the need to consider intracardiac tumors in the differential diagnosis of unexplained dyspnea, particularly in elderly patients with preserved ejection fraction and mitral inflow obstruction. Early diagnosis and surgical intervention are crucial to avoid serious complications and ensure favorable outcomes.

## Conclusions

Left atrial myxoma remains a rare but potentially life-threatening cause of heart failure symptoms due to its ability to dynamically obstruct the mitral valve. This case illustrates how progressive dyspnea in an elderly, comorbid patient may reveal an underlying cardiac mass, masquerading as valvular disease. Prompt echocardiographic evaluation, surgical management, and appropriate preoperative assessment, including coronary angiography when indicated, are essential for optimal outcomes. Despite its benign nature, left atrial myxoma warrants timely intervention to avoid embolic and hemodynamic complications. Long-term follow-up is necessary to monitor for recurrence, especially in the context of familial predisposition. This case also underscores the importance of implementing structured long-term surveillance following myxoma resection.
